# Exploring Cyclodextrin Complexes of Lipophilic Antioxidants: Benefits and Challenges in Nutraceutical Development

**DOI:** 10.3390/ijms262311682

**Published:** 2025-12-02

**Authors:** Mario Jug, Kristina Radić, Laura Nižić Nodilo, Emerik Galić, Tea Petković, Marina Jurić, Nikolina Golub, Ivanka Jerić, Dubravka Vitali Čepo

**Affiliations:** 1 Faculty of Pharmacy and Biochemistry, University of Zagreb, Ante Kovačića 1, 10000 Zagreb, Croatia; mjug@pharma.unizg.hr (M.J.); kradic@pharma.unizg.hr (K.R.); lnizic@pharma.unizg.hr (L.N.N.); egalic@pharma.unizg.hr (E.G.); tpetkovic@pharma.unizg.hr (T.P.); mjuric@pharma.unizg.hr (M.J.); nikolina.golub@pharma.unizg.hr (N.G.); 2 Ruđer Bošković Institute, Bijenička Cesta 54, 10000 Zagreb, Croatia; ijeric@irb.hr

**Keywords:** cyclodextrin, inclusion complex, antioxidant, carotenoid, retinoid, tocopherol, curcumin, capsaicin

## Abstract

Antioxidants are essential bioactive compounds widely recognized for their health benefits in preventing oxidative stress-related diseases. However, many lipophilic antioxidants suffer from poor aqueous solubility, low chemical stability, and limited bioavailability, restricting their application in food, nutraceutical, and pharmaceutical industries. Cyclodextrins (CDs), a class of cyclic oligosaccharides with a hydrophilic exterior and lipophilic interior, present an effective strategy to encapsulate and deliver these compounds by improving their solubility, stability, and therapeutic efficacy. This review critically examines the structural features and derivatives of cyclodextrins relevant for antioxidant encapsulation, mechanisms and thermodynamics of inclusion complex formation, and advanced characterization techniques. It evaluates the influence of CD encapsulation on the oral bioavailability and antioxidant activity of various lipophilic antioxidants supported by recent in vitro and in vivo studies. Moreover, sustainable preparation methods for CD complexes are discussed alongside safety and regulatory considerations. The comprehensive synthesis of current knowledge contributes to guiding the rational design and development of CD-based antioxidant nutraceuticals, addressing formulation challenges while promoting efficacy and consumer safety.

## 1. Introduction

Lipophilic antioxidants such as carotenoids, tocopherols, retinoids, curcumin (CUR), capsaicin (CAP), and coenzyme Q10 (CoQ10) are extensively studied for their crucial role in maintaining human health by counteracting oxidative stress, which is linked to numerous chronic diseases and ageing processes [1,2,3]. However, their practical application in food, pharmaceutical, and nutraceutical industries is severely limited by poor water solubility, chemical instability, and low bioavailability. These challenges call for innovative formulation strategies to enhance their stability, solubility, and therapeutic efficiency [4].

**Figure 1:**
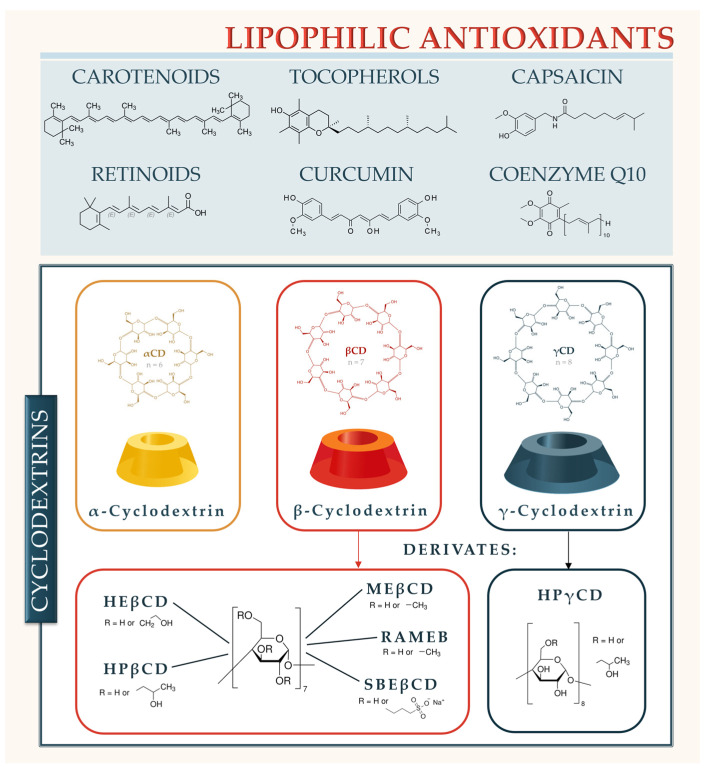
Lipophilic antioxidants and cyclodextrins analyzed in this study. HEβCD (hydroxyethyl-β-cyclodextrin), HPβCD (hydroxypropyl-β-cyclodextrin), MEβCD (methyl-β-cyclodextrin), RAMEB (randomly methylated-β-cyclodextrin), SBEβCD (sulphobuthylether-β-cyclodextrin), and HPγCD (Hydroxypropyl-γ-cyclodextrin). The present study aims to comprehensively explore the benefits and challenges associated with CD-encapsulation of antioxidants in the context of nutraceutical development focusing primarily on per os application. Namely, parenteral applications face limitations due to safety and toxicology; therefore, at present, only hydroxypropyl-beta-cyclodextrin (HPβCD) and sulfobutylether-beta-cyclodextrin (SBEβCD) are classified as safe for parenteral administration [6].

Cyclodextrins (CDs) are cyclic oligosaccharides with a hydrophilic exterior and a lipophilic cavity, making them effective carriers for lipophilic antioxidants [5]. This encapsulation not only enhances the aqueous solubility and chemical stability of these compounds but also improves their bioavailability and antioxidant efficacy. Over the past decade, advances in CD chemistry, including novel derivatives and polymeric forms, have expanded the potential of CD-based delivery systems in overcoming the intrinsic limitations of lipophilic antioxidants [5] (Figure 1).

**Figure 2:**
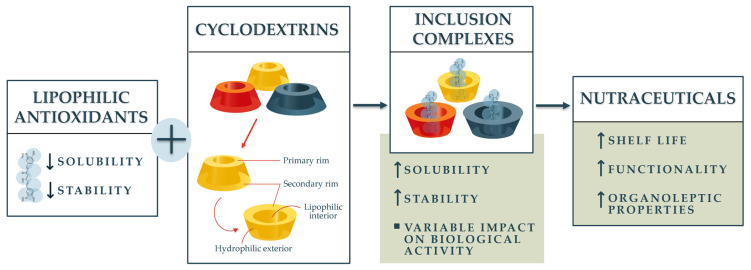
Conceptual framework of the study.

By systematically reviewing the structural features, physicochemical properties, complexation mechanisms, and characterization techniques of CDs and their inclusion complexes, this work provides critical scientific insight into optimizing formulation approaches (Figure 2). Additionally, it evaluates the biological impacts, including enhanced antioxidant activity and bioavailability, supported by cutting-edge in vitro and in vivo studies.

This article integrates recent advancements in CD technology with the specific needs of lipophilic antioxidant formulation, thereby promoting sustainable and efficient nutraceutical products. It addresses existing gaps in mechanistic understanding and practical challenges such as scalability, safety, and regulatory considerations, setting a foundation for future research and application in functional foods, dietary supplements, and therapeutic agents.

## 2. Cyclodextrins in Nutraceutical Development

### 2.1. Cyclodextrins—Structural Features and Novel Cyclodextrin Derivatives

CDs are a group of nonreducing cyclic oligosaccharides known for more than 120 years [5,7]. Natural αCD, βCD, and γCD, produced by bacterial starch degradation, are assembled of 6, 7, or 8 α-1,4-linked D-glucopyranose units, respectively. Recently, small CDs consisting of 3 and 4 glucopyranose units were synthesized [8], while large CDs with more than 8 glucopyranose units have been known for some time [9] but are not relevant for nutraceutical and drug delivery. CDs have a hollow truncated cone structure, with hydroxyl groups oriented to the exterior of the molecule, giving it a hydrophilic character. Primary hydroxyl groups are directed toward the narrower edge of the cone, while secondary ones are located on the broader rim of the molecule. The central cavity of the CD is lipophilic, presenting the same polarity as diluted ethanol, and its size is determined by the number of glucopyranose units (Table 1) [10,11,12].

Inter-and intra-molecular hydrogen bonding between vicinal C2 and C3 hydroxyl groups of adjacent glucopyranose units diminishes CD’s ability to form hydrogen bonds with the surrounding water molecules and contributes to a highly stable crystal lattice formation. Because of that, natural CDs, and especially βCD, are less soluble in water than corresponding linear maltodextrins. Substitution of any of the hydroxyl groups in the CD molecule disrupts the hydrogen-bond formation and results in a dramatic increase in the CD solubility. Numerous CD derivatives with increased solubility improved functionality, and a more acceptable toxicological profile with respect to that of natural CDs have been synthesized [7,11,18]. However, to gain optimal solubility and complexation ability, the average number of substituents per glucopyranose unit should be low [8]. The list of pharmaceutically most important CDs is given in Table 1; however, novel CD derivatives are now emerging. Among them, thiolated CDs, as mucoadhesive compounds able to prolong gastrointestinal residence time [18,19], and a wide selection of polymeric CD derivatives, like CD-based polyrotaxanes, grafted CD polymers, crosslinked CD polymers etc., that are stimuli-responsive and are able to form well-defined aggregated nanostructures [20] are the most prominent.

### 2.2. Metabolism and Safety of Cyclodextrins

After oral administration, γCD and its derivatives are completely digested in the gastrointestinal tract (GIT) by salivary and pancreatic α-amylases. In contrast, αCD, βCD, and their hydrophilic derivatives are resistant towards α-amylases and are dominantly digested by bacteria in the colon. Furthermore, high molecular weight, numerous hydrogen bond donors, and acceptors in the CDs’ structure, as well as pronounced hydrophilicity, restrict their permeability across biological membranes by passive diffusion [7,12]. All this contributes to the limited oral bioavailability of CDs in animals and humans, ranging from 0.1 to 3% of the applied dose, so they are practically nontoxic when administered orally. The maximum recommended oral daily doses of α-cyclodextrin (αCD), β-cyclodextrin (βCD), γ-cyclodextrin (γCD), and hydroxypropyl-β-cyclodextrin (HPβCD) are 6 g, 0.5 g, 10 g, and 8 g, respectively. Administration of higher doses may result in diarrhea, attributable to the osmotic activity of CDs [21]. An interaction between orally ingested CDs and the absorption of fat-soluble vitamins or other lipophilic nutrients is not anticipated, as the formation of inclusion complexes is reversible. Furthermore, γCD is readily digested in the small intestine, and studies with βCD, a non-digestible CD, have shown that the bioavailability of A, D, and E vitamins is not impaired [15]. RAMEB, a more lipophilic derivative, shows an oral bioavailability of 12% in rats. Both αCD and βCD are unsuitable for parenteral administration due to renal toxicity, while among CD derivatives, methylated CDs as surface-active agents cause significant hemolysis [10]. HPβCD, SBEβCD, γCD, and HPγCD are considered safe even for parenteral administration, displaying a small volume of distribution and rapid clearance in intact form by glomerular filtration. Pharmacokinetic studies show that more than 90% of parenterally administered HPβCD and SBEβCD are eliminated from the body within 6 h, and over 99.9% within 24 h, posing no risk of accumulation in individuals with normal kidney function. However, in patients with severe renal impairment (renal creatinine clearance < 10 mL/min), CD accumulation will be observed, but its clinical significance is still to be determined [7,22]. Administration of natural and hydrophilic CDs on nasal, buccal, vaginal, or rectal mucosa is safe in a wide range of concentrations, while for RAMEB, the concentration (~5%) and exposure time should be limited [10,23]. Natural CDs and their hydrophilic derivatives are not able to permeate skin in significant amounts (0.02% and 0.3% of the applied dose for HPβCD and RAMEB, respectively) and are generally considered to be non-irritant to the skin. However, under occlusive conditions, transdermal absorption of CDs could be more pronounced [21].

### 2.3. Regulatory Status

The regulatory status of CDs has continuously developed over the past 40 years [11]. Natural αCD, βCD, and γCD are listed in the Generally Regarded as Safe list of the Food and Drug Administration (FDA) for their use as food additives and, along with HPβCD, have monographs in the European Pharmacopeia and the United States Pharmacopeia and the National Formulary (USP/NF). SBEβCD has a monograph only in USP/NF, while other CD derivatives are considered related substances. The main problem with CD derivatives from a regulatory standpoint relates to the homogeneity of the final product. For example, methylated βCD derivatives are commercially available in various qualities, with isomeric purity of 50%, 80%, or 95%, but there is no 100% pure 2,6-dimethyl-βCD, making it difficult to set the quality standards [23]. In the European Union, βCD is approved as a food additive (E459), with an acceptable daily intake of 5 mg/kg/day [11,21], and αCD is regulated as a dietary fibre with an authorized health claim “*for contributing to the reduction of postprandial glycemic responses*” [24]. Nowadays, in drug formulations, CDs are considered as excipients and not as part of the drug substance. Thus, βCD, HPβCD, SBEβCD, γCD, and HPγCD are introduced into the FDA’s list of Inactive Pharmaceutical Ingredients [7] but have not yet been recognized as ingredients for the use in food [25]. In the context of a new drug application, when a given CD derivative is used for the first time in humans, it is treated as an active ingredient. A complete safety evaluation may not be needed if a given CD derivative has been previously used at the same concentration and route of administration as in the marketed product [11].

### 2.4. Inclusion Complex Formation

An inclusion complex is formed by the reversible entrapment of a poorly soluble compound or, more often, a sterically compatible lipophilic functional moiety of the molecule into the lipophilic CD central cavity. This causes the displacement of energy-rich water molecules from the CD central cavity, accompanied by a whole set of intermolecular drug/CD interactions, including hydrogen bonding, van der Waals forces, and hydrophobic interactions as well as ionic interactions (in case of charged drugs and CD derivatives), all contributing to steric and thermodynamic stabilization of the system. Therefore, inclusion complex formation occurs spontaneously in the aqueous media, and no covalent bonds are formed or broken during the inclusion complex formation [10,11,26]. Inclusion complex formation is a reversible process characterized by a dynamic equilibrium between the association and the dissociation of the drug/CD complex, described by inclusion complex association (i.e., stability) constant, ranging from 2 M^−1^ (fenofibrate) up to 40,000 M^−1^ (telmisartan) [27]. One molecule is usually included in one CD central cavity, presenting 1:1 molar stoichiometry. But in certain cases, higher-order complexes were described, where one drug molecule is included in two or more CDs, depending on the drug geometry [28].

Natural CDs self-assemble in an aqueous solution, forming aggregates in a concentration-dependent manner [29,30,31]. Aggregation is further promoted by the inclusion complex formation and chemically modified CDs, which alone have a low affinity for aggregation but self-assemble upon inclusion complexation with lipophilic drugs due to the surfactant-type structure of the complex formed [30]. CD aggregation should be considered in the development of liquid formulations containing high CD concentrations, as it could affect the inclusion complex formation, the physicochemical properties, and the in vivo performance of such products [32].

Absorption through the biological membrane and binding to the targeted receptor site are prerequisites for the drug/nutraceutical’s biological action. Therefore, when administered as an inclusion complex, the included compound must first be released from the complex formed. CDs will restrict compound permeation across biological membranes due to their high molecular weight and hydrophilic character, and they will sterically hinder their binding to their targeted sites. The compound release from the inclusion complex is mainly governed by simple media dilution, usually after oral or parenteral administration. Furthermore, other mechanisms can shift the equilibrium toward complex dissociation, such as the compound binding to proteins, competitive displacement from the CD cavity caused by bile salt in the GIT, and direct drug partitioning from the complex to the tissue. All this results in rapid and complete drug release from the complex after parenteral and oral administration, so CD complexation will not hamper its therapeutic effect. CD complexation will affect the drug pharmacokinetics after parenteral administration only in cases where the binding constant is higher than 100,000 M^−1^. In topical applications, media dilution is limited, and CDs can hamper drug release and absorption. Therefore, the use of excessive CD concentration in topical formulations should be avoided [11,28,33].

Inclusion complex formation enhances solubility, dissolution rate, chemical stability, and bioavailability of encapsulated molecule [7,34]. Thereby, CD complexation can be considered multifunctional technology to increase therapeutic potential and efficiency of drugs, especially as it could reduce or prevent certain side effects of the drugs [35], enhance drug permeation across the biological membranes [11,36], prevent drug-to-drug and drug-to-excipient interactions [37], favourably modify organoleptic characteristics of the included molecule [38], and even convert liquid and volatile compounds to technologically more acceptable free-flowing powders [39]. Because of such multifunctionality, CDs emerge as attractive excipients for the development of novel pharmaceuticals, including the reformulation of existing drug formulations and enabling an alternative application route for a given drug [28]. In some cases, the addition of polymers, organic acids, metal ions, or lipids may further enhance CD performance by a ternary or higher-order complex formation [11]. Presently, CD technology has been employed in the development of more than 120 pharmaceutical products available worldwide, while numerous formulations are in advanced phases of clinical testing [17].

In addition to pharmaceutical application in drug formulation development, certain CDs are used in the food industry to solubilize and/or chemically stabilize different food ingredients, remove unwanted or harmful food components like cholesterol or mycotoxins, improve antioxidant efficiency, mask unpleasant taste, improve sensory quality, preserve the colour of food, and are applied in food packaging [25,40,41].

### 2.5. Preparation and Characterization of Cyclodextrin Complexes—Sustainable Approaches

The development and preparation of CD complexes is a complex task requiring the simultaneous use of different preparation and characterization techniques to define optimal CD derivative and the preparation conditions for the molecule of interest. The usual approach is starting from the solution, using the phase solubility studies, where the effect of CD concentration on the drug solubility is assessed, usually by UV/VIS or HPLC analysis [38]. This phase aims to determine the type and concentration of the CD required to obtain the maximum solubilization of the investigated compound. This approach also enables the calculation of the association constant for each drug/CD combination. However, this step is rather time-consuming, as it should be performed in different media like water and other biorelevant buffered media, depending on the characteristics of the studied compound, such as solubility, chemical stability, pKa, etc. Depending on the obtained results, CD complexation efficiency and drug solubilization should be further enhanced by pH regulation, the addition of co-solvents, or by the formation of ternary complexes with suitable compounds, like amino acids, polyhydric acids, and polymers [7]. This, in turn, requires the development and validation of adequate drug quantification methods for each of the selected media. An additional problem arises with poorly soluble drugs, whose concentration in the samples without CDs may fall below the limit of quantification of the method employed. In the case when drug quantification is performed by HPLC, which is nowadays a gold standard, a large volume of solvents used as the mobile phase should be considered, requiring strict hazardous waste management. In this sense, capillary electrophoresis, as a more environmentally friendly method for drug/nutraceutical quantification, should also be employed [42]. Furthermore, molecular modelling methods, including molecular docking, molecular dynamics, and free energy binding analysis, may provide a helpful shortcut in selecting the optimal CD derivative to solubilize the compound of interest, significantly reducing the experimental time and waste production [43].

Depending on the chromophore presence in the structure of the examined molecule, the inclusion complex formation may change UV absorption spectra, enhance fluorescence, or lead to changes in circular dichroism spectra, providing additional analytical techniques to monitor drug/CD interactions in the solution [44]. Finally, perhaps the most important analytical technique to characterize the CD complexation in the solution is NMR spectroscopy, which provides direct evidence of the actual inclusion complex formation by observation of the chemical shift difference in proton (^1^H NMR) or carbon (^13^C NMR) between free guest and host species and the presumed complex. Furthermore, the measurement of chemical shift changes in the guest as a function of increased CD concentration (i.e., NMR titrations or Job’s plot) enables the determination of stoichiometry of the formed CD complex. Finally, two-dimensional NMR techniques like NOESY (Nuclear Overhauser Effect Spectroscopy) and ROESY (Rotational Overhauser Effect Spectroscopy) evidence the spatial proximity of host and guest protons, enabling precise determination of the drug/CD binding mode [44,45].

The other useful techniques to characterize the complex formation in the solution are dynamic light scattering, enabling monitoring of aggregation phenomena of CDs and complexes formed [31], and isothermal titration calorimetry, useful to precisely determine thermodynamic parameters of the complexation process, like complex stoichiometry, association constants, as well as enthalpy and entropy changes upon complexation in a single experiment [46,47].

After selecting the most suitable CDs for the compound of interest, a complex in solid state should be prepared. It must be underlined that demonstrating the actual inclusion complex formation in the solution does not automatically guarantee its existence in the solid state. Because of this, the complex preparation method should be carefully selected and optimized based on the characteristics of the drug and CD and the desired characteristics of the obtained product [47].

The traditional approach involves methods for the complex preparation in the solution. Here, the drug and CD are separately dissolved in adequate media. The critical properties are solubility and chemical stability of both drug and CD, directing the selection of the solvents for the complexation media and the drying technique employed for isolating the complexes in the solid state. For the drug, this mainly involves using organic solvents that are miscible with water. However, caution must be taken regarding the toxicological profile of the solvent used, as it may remain in the final product. This will be especially pronounced in the case of solvents having a high affinity for complexation with CDs, thereby competing with the drug for the inclusion complex formation. Because of that, the list of available solvents is mainly restricted to ethanol [35]. CDs are usually dissolved in water, and the complex formation occurs by mixing the ethanolic drug and aqueous CD solution. The resultant mixture may be further stirred, sonicated, or heated to promote drug/CD interaction. Heat must be used prudently, as excessive heat may lead to chemical degradation of the drug and/or inclusion complex dissociation due to high thermodynamic activity in the system at temperatures above 50 °C. In some cases, the drug may be dissolved directly in the CD aqueous solution. Also, other approaches like pH modification and salt formation may be beneficial in this regard, depending on the physicochemical characteristics of the drug. After the inclusion complexation is achieved in the solution, the solvent must be removed to isolate the CD complex in the solid state [37,38]. In the case of natural CDs, this may be obtained by precipitation or using other drying techniques like spray-drying and freeze-drying. Both techniques are suitable for the industrial production of CD complexes. The spray-drying technique is particularly suitable, as it ensures rapid solvent removal, thus preventing the dissociation of the complexes formed. Indeed, slow solvent removal in rotational vacuum evaporators may lead to complex dissociation, especially when the included compound has a very stable crystal lattice [48]. The drawback of the spray-drying technique is the fact that it is not suitable for thermolabile and volatile compounds, and the product yield is typically lower (40–79%), as some product particles are lost through the exhaust of the instrument or are attached to the walls of the drying chamber and cannot be collected [49]. Freeze-drying is a method of choice when dealing with CD complexes of the thermolabile compounds; however, in this case, the organic solvent (i.e., ethanol) must first be removed, as it interferes with the freezing step of this drying process. Finally, long duration and high energy consumption are other drawbacks of the freeze-drying process [50]. In this regard, new, more environmentally friendly, and sustainable methods must be considered for preparing CD complexes from the solution. One such method, preparing complexes using supercritical CO_2_, has great potential owing to its mild critical point that ensures the chemical stability of the drug, tunable solvent power, and the absence of solvent residue after depressurization [51]. Furthermore, the potential of green solvents, like ionic liquids and deep eutectic solvents as the complexation media should be further examined, as they provide an interesting opportunity to address the problem of the limited solubility of native CDs (i.e., the solubility of βCD may reach 1000 mg/mL at 20 °C) [52,53].

Kneading is a semi-solid state-based method where a drug/CD mixture is wetted with a small amount of water or water/ethanol mixture until the paste-like product is obtained. Such a product is then mixed (i.e., kneaded) for some time to promote drug/CD interaction. Finally, the added solvent is removed by drying at a reduced pressure, and the resultant product is milled and sieved. This method is usually used for compounds with limited solubility and results in the low content of the actual drug/CD complex [42].

Rising environmental awareness, the need for sustainability, and the implementation of a green chemistry approach are setting the focus on solid-state methods for the preparation of CD complexes. These methods eliminate problems related to the low solubility and chemical instability of drugs in solution, as well as the potential toxic effects of residual solvents. In this light, mechanochemical activation by grinding emerged as a rapid, highly efficient, solvent-free, sustainable approach [54,55]. Here, the complexation is obtained in high-energy vibrational or rotational mills, where the energy of the colliding balls (grinding bodies) is transferred to the treated material (equimolar drug/CD mixture) upon impact. This first causes the shredding of the treated materials up to a critical threshold, accompanied by intense mixing. Further energy supply by grinding leads to amorphization of the material and, consequently, its chemical activation, enabling drug/CD complexation in the solid state. However, parameters of the grinding process, like grinding frequency, time, and temperature at which the process is performed, must be carefully optimized to avoid degradation of the treated material [56]. Furthermore, the grinding process may raise unconventional supramolecular interactions that lead to novel molecular arrangements not observed in the solution, providing significant advantages in the formulation development [57].

It has been shown that the technique of CD complex preparation has an essential role on the physicochemical properties and the performance of the obtained product [47]. Furthermore, a comprehensive characterization of the solid product obtained is essential. This enables the selection of the most appropriate preparation technique and optimal technological parameters of the method applied to achieve the most effective drug/CD solid-state interaction in the product obtained, obtaining the desired increase in functionality [47,58].

Differential scanning calorimetry (DSC) is the most widely applied technique for characterizing CD complexes in the solid state [47,59]. This technique compares the thermal properties (most often melting temperature and fusion enthalpy) of the pure compounds (i.e., CDs and the drug of interest), their physical mixtures, and intended complexes. In general, reduction in the fusion enthalpy, shifting of the drugs’ melting peak to lower temperatures, or its complete disappearance is usually taken as an indication of the CD complexation in the solid state. However, it must be considered that the disappearance of the melting peak in the DSC thermogram of the analyzed product only indicates the drug amorphization that may be a consequence of many different non-inclusion phenomena during the CD complex preparation like fast solvent evaporation that disabled the crystal lattice formation or solid dispersion formation [60]. Also, heat-induced drug interaction brought up by thermal energy supply must be considered. Methylated CD derivatives appear especially prone to such interactions [48,61]. Because of that, DSC data must be further supported by complementary techniques like X-ray powder diffraction (XRPD). XRPD requires low sample quantity, is a non-destructive technique, and does not change the physicochemical properties of the sample during the spectra recording, providing additional insight into the solid state of the sample prepared. However, the sensitivity of XRPD in detecting the residual crystalline drug samples in the analyzed product is usually lower than that of DSC [62]. Regardless, the reduced crystallinity or complete amorphization of the drug in the sample observed by DSC and XRPD only indicates inclusion complexation. The only technique able to demonstrate the actual inclusion complex formation in the solid state is solid-state NMR (ssNMR). In particular, ssNMR can provide information on the orientation of the guest molecule inside the cavity and the complex stability in the solid state. Furthermore, it enables quantitative analysis of the phases, especially the complexed and non-complexed guest molecules. In addition, this technique allows for studying the local molecular dynamics of guest molecules and the nature of intermolecular interactions between the host and the guest [63]. The most widely applied ssNMR technique for the characterization of the CD complexes is the ^13^C cross-polarization magic-angle spinning (CP-MAS) analysis. However, this technique is somewhat laborious and expensive, requires long experimental time to obtain good-quality results, and signal assignment and data interpretation may be difficult, especially in the case of complexes of chemically modified CD derivatives [61].

Other techniques used for characterizing CD complexes in the solid state are Fourier transform infrared spectroscopy (FTIR), which provides insight into the chemistry of the solid-state interaction among included compounds and CDs, and scanning electron microscopy (SEM), a surface-imaging technique that detects changes in the sample morphology upon complexation with CDs [47]. Also, the drug content in the product obtained must be analyzed, as too harsh conditions of complex preparation (e.g., drying at too high temperatures or prolonged grinding at high grinding frequencies) may lead to drug degradation [56,64]. Furthermore, moisture, the content of residual solvents, and elemental impurities (for the complexes prepared by grinding) must be monitored, as they may impair the chemical stability and safety of the CD complexes [65]. Finally, there is a whole set of different functional tests, such as in vitro dissolution, permeation across biomimetic membranes or cell monolayers, antioxidant activity, particle size distribution, real-time and accelerated stability testing, pharmacokinetic studies etc., that enable the determination of the benefit obtained by CD complexation of the molecule of interest [33,61,64,66,67,68].

## 3. Antioxidants in Functional Foods and Nutraceuticals

### 3.1. Antioxidants and Their Mechanisms of Action

Antioxidants are compounds that can stop or slow down the oxidation of proteins, lipids, carbohydrates, and DNA in biological systems [69]. Superoxide dismutase (SOD), glutathione peroxidase, and catalase are examples of enzymatic antioxidants, while nonenzymatic antioxidants include glutathione, uric acid, alpha-lipoic acid, ubiquinones, bilirubin, metal chelators, vitamin C, carotenoids, tocopherols, and phenolics. Additionally, carotenoids, polyphenols, and tocopherols are examples of exogenous antioxidants, and the same should be sourced from the diet or supplementation [70]. Reducing oxidative stress and DNA damage are two of the primary benefits of antioxidants. In general, antioxidants work by interrupting chain reactions, mending damaged biomolecules, inactivating oxidants by adding H+, or transforming them into weaker molecules. Furthermore, they exhibit their effects by blocking lipid peroxidation chain reactions, accumulating reactive oxygen species, preventing lipid peroxidation, and actively participating in the transformation of peroxides into nonradical compounds [69]. Since antioxidants are essential for preserving health and preventing chronic diseases [71], the mechanisms of action by different groups of lipophilic antioxidants are shown in the following part.

Carotenoids are widespread natural pigments classified as bioactive compounds. They contribute to the yellow, orange, and red coloration of fruits and vegetables, such as tomato, carrot, apricot, and pepper, and the pigmentation of dark-green leafy vegetables, like spinach and kale [72]. Recognized as health promoters, carotenoids play an important role in human health and diet [73]. They reduce oxidative damage by capturing free radicals [74]. The same compounds play a significant part in the lipid oxidation process, scavenging singlet oxygen, peroxyl, sulfonyl, and NO_2_ radicals as well as providing defence against hydroxyl and superoxide radical attacks [70]. Furthermore, lycopene can scavenge superoxide radicals (LOO•) and singlet oxygen (^1^O_2_) [75]. It raises the concentrations of enzyme antioxidants as glutathione peroxidase, catalase, and superoxide dismutase. As a result, the antioxidant response element is activated, which is connected to nuclear factor E2 [76]. Additionally, non-enzymatic antioxidants like vitamins C and E can be renewed by lycopene. The cellular antioxidant defence system benefits from this [77], and it has been shown that lipids and DNA are two vital bodily components that lycopene can shield because of its antioxidant properties [78]. Furthermore, by immediately squelching reactive oxygen species, as well as by promoting glutathione synthesis in human retinal pigment epithelial cells, zeaxanthin demonstrates its antioxidant properties.

Vitamin E encompasses two families of lipophilic compounds: tocopherols and tocotrienols. Each family comprises four stereoisomers (α, β, γ, and δ), giving rise to eight distinct forms of vitamin E. Tocopherols are characterized by a saturated phytyl tail, whereas tocotrienols contain an unsaturated isoprenoid side chain, conferring unique biophysical and pharmacological properties to each isoform [79]. In typical diets, γ-tocopherol predominates, whereas most commercially available supplements deliver α-tocopherol, selected for its superior bioavailability and for establishing dietary reference intakes.

The primary role of vitamin E is to terminate lipid peroxidation by scavenging chain-propagating peroxyl radicals in cell membranes, thereby preventing oxidative damage to lipids, proteins, and DNA. It protects against lipid and lipoprotein peroxidation by acting as a potent scavenger of superoxide, peroxyl, and hydroxyl radicals [74,80]. Additionally, it has outstanding scavenging capabilities for reactive nitrogen species [81]. Beyond its classical antioxidant mechanism, individual tocopherols and tocotrienols exhibit divergent activities, including modulation of signal transduction pathways, gene expression, and inflammatory responses [82], which makes it an effective nutritional supplement and antioxidant with a wide range of potential in the food, pharmaceutical, and cosmetic [83]. Daily intake of this antioxidant will aid in raising superoxide dismutase levels [84] and promote the activity of several anti-oxidative enzymes, including peroxidase and catalase [80].

The ability of retinoids to quench ^1^O_2_, their high molar absorption coefficient, and their ability to lose protons when interacting with reactive species to form a less reactive radical centre stabilized by the polyene network are the three main ways that they shield cellular components from damage caused by photooxidation and reactive oxygen species (ROS) [85].

Retinoids include a family of molecules containing a 20-carbon structure with various chemical groups at the 15 carbon position. Variations at the 15 carbon position yield different retinoid forms, including retinol, retinal, retinoic acid, and retinyl ester. The most potent form of vitamin A, all-trans-retinol, is the form of retinol in the diet. It reverses signs and symptoms of vitamin A deficiency and is the standard for vitamin A activity [86]. Either pre-formed vitamin A from animal products (retinol) or provitamin A carotenoids from plant products is an acceptable vitamin A source, although provitamin A carotenoids must be consumed in larger quantities [87]. All-trans retinoic acid is the active form of vitamin A in almost all biological processes.

Retinoic acid regulates the expression of various genes that encode for structural proteins, such as keratins in the skin; enzymes, such as alcohol dehydrogenase; extracellular matrix proteins, such as the basement membrane protein laminin; and retinol binding proteins and receptors [88]. Vitamin A also regulates genes, including those of the dopaminergic system, in the brain, and central nervous system [89], and acts as a cofactor in mucopolysaccharide synthesis, cholesterol synthesis, hydroxysteroid metabolism, and glycoprotein glycosylation. Retinoids exhibit antioxidant activity primarily through their chemical structure, characterized by multiple conjugated double bonds that enable the quenching of ROS such as singlet oxygen and peroxyl radicals. This antioxidant action involves scavenging, inactivation, and termination of oxidative radical chain reactions, thereby protecting cellular components from oxidative damage. Mechanistically, retinoids stabilize free radicals by forming less reactive radical centres, aided by their polyene network. Additionally, retinoids influence antioxidant pathways by modulating enzymes such as SOD, catalase, and glutathione peroxidase, enhancing cellular defence against oxidative stress. These properties are important in various physiological contexts, including skin photoprotection and the mitigation of oxidative stress in disease states. However, the antioxidant effects can be complex, as retinoids may also induce oxidative stress under certain conditions, depending on dose and cellular context [85]. Vitamin A used in clinical research has included oral, topical, and injectable products. Most dosing recommendations for vitamin A are for pre-formed vitamin A (retinol). Vitamin A is required for vision, growth and bone development, reproduction, cell proliferation and differentiation, immune function, and the integrity of mucosal and epithelial surfaces.

CoQ10 is naturally found in all of our body’s cell membranes as the essential part of the mitochondria’s oxidative phosphorylation process [90]. Additionally, it is ideally situated near the unsaturated lipids in membranes, serving as a major scavenger of free radicals to prevent lipid peroxidation and shielding DNA and cellular membranes from oxidative damage [91]. Additionally, by maintaining nitric oxide, CoQ10 can enhance blood flow and protect blood vessels [90].

CUR exhibits potent antioxidant activity primarily due to its chemical structure, which contains phenolic hydroxyl groups and a conjugated diketone moiety. These structural features enable CUR to donate hydrogen atoms to neutralize ROS and free radicals, thus functioning as a chain-breaking antioxidant [92,93]. CUR enhances systemic indicators of oxidative stress by modifying the activity of the catalase, SOD, and GSH (glutathione), which are involved in the neutralization of free radicals [94]. It inhibits enzymes that produce ROS, including xanthine hydrogenase/oxidase and lipoxygenase/cyclooxygenase [95].

CAP exhibits significant antioxidant activity mainly through its free radical scavenging mechanisms. It primarily acts by hydrogen transfer (HT) from its phenolic OH group to neutralize ROS, effectively scavenging peroxyl and alkoxyl radicals and reducing lipid peroxidation in cellular membranes, mitochondria, and lipoproteins. This antioxidant effect helps prevent oxidative damage by preserving endogenous antioxidants like glutathione and restoring the activity of enzymes such as superoxide dismutase and glutathione reductase. CAP’s antioxidant capacity can surpass that of vitamin E, melatonin, and caffeine in some models, and its action is often mediated through the activation of the TRPV1 receptor and the Nrf2 signalling pathway, which enhances cellular defence mechanisms against oxidative stress. The aromatic benzene ring and the ortho-position methoxy and hydroxy groups in CAP’s structure play a crucial role in its radical scavenging efficacy. These combined mechanisms contribute to its protective effects against oxidative stress-related damage and inflammation in biological systems [96,97].

Table 2 presents the most important human intervention randomized clinical studies or meta-analyses performed in the past 10 years that investigated potential health effects that can be achieved by oral supplementation of lipophilic antioxidants indicating their relevance in contemporary clinical practice.

Depending on the antioxidative mechanisms explained above, intake of carotenoids has shown positive benefits on cardiovascular, cognitive, and visual health. The two carotenoids that were evaluated the most in the clinical trials were lutein and zeaxanthin, compared to the β-carotene supplementation that is rarely studied as a single molecule [137]. Patients who took lutein supplements experienced a substantial increase in macular pigment optical density, according to the meta-analysis. The mentioned effect was determined by the supplementation in a daily dose of 20 mg for a period longer than 6 months [101]. Furthermore, lutein has been shown to improve cognition in both young and older adults [98]. According to Table 2, the improvement in visual memory and performance by lutein and zeaxanthin supplementation was also determined in a few other studies, in a recommended dose of 10 mg of lutein and 2 mg of zeaxanthin for six months [105]. Additionally, lutein has positive benefits on other tissues, including the brain, where it has been linked to enhanced cognitive function [138], but can also reduce skin inflammation, inhibit oncogenesis, and increase tolerance to UV exposure. Lycopene may be useful in the treatment of cardiovascular disease, according to studies by [109,110]. In a placebo-controlled, double-blinded, randomized study, performed by Grether-Beck and co-authors [103], three months of consumption of 5 mg of lycopene daily resulted in the protection of human skin against UV radiation. Wolak and co-authors [111] demonstrated efficacy in lowering systolic blood pressure in the hypertensive subject group. Supplementation with astaxanthin was monitored in a dose range from 2 to 12 mg up to six months.

A total of 400 IU daily was the dose of oral supplementation with vitamin E used in different human clinical studies. For example, Jaffary and co-authors [117] determined effectiveness with the four-month supplementation of patients with atopic dermatitis, without side effects. Vitamin E, a potent antioxidant, can help to safely alleviate clinical symptoms and lower oxidative stress conditions in people with late-stage knee osteoarthritis. The above-mentioned impact was determined following the two-month supplementation with 400 IU daily [118]. Moreover, it has been shown that oral retinoids, especially isotretinoin, are a successful treatment for acne vulgaris [139]. Isotretinoin has been indicated as a successful treatment for this inflammatory condition, particularly in instances that have left scars or have not responded to previous treatments. In that context, Dhaked and co-workers [121] have determined 20 mg for six months as an efficient therapy for mild to severe acne vulgaris. An evaluation of the pertinent literature indicates that oral isotretinoin is superior to oral antibiotics in achieving long-lasting remission following treatment [140].

CAP supplementation during high-intensity exercise could be employed to enhance muscular endurance and improve exercise performance in physically active adults in a dose of 12 mg 45 min before exercise [123,124,125].

CUR efficiency in the improvement of endothelial function was shown in the double-blinded randomized studies by Oliver and co-workers and Santos-Parker and co-workers [126,127], with the dose of 200–400 mg daily for 8–12 weeks. Furthermore, the potential antioxidative and anti-inflammatory effects of the CUR supplementation is shown in Table 2.

The beneficial effect of the oral CoQ10 supplementation was shown for a dose range from 100 to 600 mg daily, usually during a one-month to one-year period. Potential efficiency in the prevention of migraines and reduction in the frequency of headaches was detected by the consumption of 400 mg daily for three months, while a dose of 500 mg daily for one year relieves symptoms of depression and fatigue in patients with multiple sclerosis [90,134,136].

### 3.2. Bioavailability of Lipophilic Antioxidants

Oral administration is usually the recommended route of application for bioactive compounds with therapeutic or preventive advantages when patient compliance is essential. However, the hostile environment of the GIT can decrease the solubility, stability, and absorption of orally ingested bioactive substances [141]. Specifically, compounds with low water solubility do not dissolve easily in the GIT, and gastrointestinal barrier blocks penetration into the systemic circulation. Additionally, chemical and enzymatic degradation might decrease the quantity that is accessible for absorption [142]. In that context, antioxidant structure, half-life, interactions with other compounds, and delivery mechanisms all affect antioxidant bioavailability. Bioavailability refers to the portion of the compound that is absorbed into the bloodstream, distributed through the systemic circulation, exerts its post-metabolic biological effects, and is then eliminated [143]. Carotenoids’ low water solubility and vulnerability to light- and oxygen-driven deterioration limit their bioactivity [144]. Absorption can range from 5 to 50% and varies greatly based on other dietary components included in the meal [145]. Lycopene, as one of the main serum carotenoids found in human blood and tissues, has a half-life of two to three days within the body and a human plasma concentration that varies between 0.22 and 1.06 nmol/mL [146]. It has been shown that factors influencing lycopene bioavailability include cooking, food cutting or chopping, dosage, interactions of lycopene with other carotenoids, the presence of fats or oils, and the physiological state of the individual consuming the lycopene [147]. According to reports, lycopene is only 23% soluble when combined with oil and can reach 5% when ingested with tomato products or juice [148]. On the other hand, the usage of tocopherols is limited not only because of their low bioavailability, poor water solubility, and inconsistent absorption [149] but also stability influenced by exposure to light, oxygen, and high temperatures [150]. After 3–4 h, peak plasma levels of α-tocopherols are reached, whereas those of γ and δ tocotrienols are reached after 5 h of consumption [151]. Because of its higher binding affinity with the hepatic α-tocopherol transfer protein, only α-tocopherol has high levels in plasma and tissues among all isoforms. This allows it to remain in plasma for an extended period of time to exert its pharmacological effects, whereas other tocopherol isoforms are quickly metabolized and eliminated in feces [152]. Furthermore, isotretinoin is almost insoluble in water and extremely lipophilic. Even though the molecule easily passes across cell membranes, oral bioavailability of isotretinoin is limited due to poor solubility in the intestinal fluid environment [153]. Due to low water solubility, poor intestinal permeability, and hepatic metabolic biotransformation, over half of an oral dose of CUR is eliminated undisturbed in the feces, and very little CUR is detectable in plasma following low-dose supplementation [154,155]. According to research on humans, the peak plasma concentration of CUR is only 11.1 nmol/L 1–2 h after 3.6 g of CUR is consumed [156]. CoQ10’s bioavailability is also limited and is usually in the order of 5% or less, due to its unique chemical structure and poor water solubility [157,158].

In order to overcome the constraints of peroral administration and enhance the therapeutic effect resulting from poor stability/bioavailability, lipophilic antioxidants can be integrated into a biocompatible substrate to improve their stability, half-life, tissue-specific distribution, and bioavailability [91]. In that context, a lot of research has indicated that the encapsulation of antioxidants can result in advantageous effects, protection of the bioactive principle, controlled release, a reduction in the amount of biocompound needed, and increased antioxidant efficacy before intake [159,160,161,162].

### 3.3. Challenges in Formulating Lipophilic Antioxidants

The most common antioxidant groups, including carotenoids, tocopherols, and retinoids, comprise highly hydrophobic molecules which, with their aqueous solubility of around 0.01 mg mL^−1^, are considered practically insoluble [163,164]. As explained previously, it contributes to their low bioavailability, which is further affected by other factors such as food processing, meal composition, and interactions within the gastrointestinal system [165,166]. Furthermore, exposure to high temperatures, oxygen, and/or light may hinder the antioxidants’ stability, considering their susceptibility to chemical degradation in suboptimal conditions [167,168]. To overcome these challenges, many strategies are being employed, including optimizing the processing conditions, chemical and physical modification, and incorporating the molecules in various formulations, mostly lipid-based delivery systems [169]. The most commonly developed antioxidant-loaded delivery systems for food application are lipid-based nanosystems, including vesicular systems such as liposomes and niosomes, as well as solid lipid nanoparticles (SLNs), nanostructured lipid carriers (NLCs), and nanoemulsions [170].

Liposomes are spherical nanoparticles composed of one or more phospholipid bilayers, whereas niosomes usually comprise synthetic, non-ionic surfactants, usually with only one hydrophobic chain [167,171]. Due to the amphiphilic nature of these constituents, both liposomes and niosomes may be used for the encapsulation and delivery of both hydrophilic and hydrophobic molecules [167]. In food industry, liposomes are mostly used as texture- and water retention-modulators, but recent trends lean towards utilizing liposomes for encapsulation of sensitive food molecules, including antioxidants such as resveratrol, CUR, β-carotene, and apigenin [172]. Similarly, niosomes are also employed for lipophilic antioxidants encapsulation [173] but are characterized by the lower production cost, simpler preparation methods, as well as a better stability profile compared to liposomes [171].

Lipid nanoparticles may be categorized depending on the state of matter of the comprising lipids: SLNs are nanoparticles, which consist of lipids in solid form at both physiological and storage/room temperature, dispersed in water, and stabilized by one or more surfactants [166], while NLCs comprise the combination of liquid and solid lipids, creating a less-ordered structure. Both of these nanosystems are under investigation for food optimization and lipophilic compound encapsulation, mainly carotenoids, tocopherols, and polyphenols [174].

Nanoemulsions used for the enhancement of delivery of lipophilic antioxidants in nutrition products are almost exclusively oil-in-water (*o*/*w*) emulsions, defined as colloid aqueous dispersions in which nanoscale oil droplets are stabilized by emulsifying agents such as surfactants and polymers [167]. So far, their use has been investigated for the incorporation of essential oils, alone or in combination with lipophilic bioactive compounds such as carotenoids, tocopherol, or retinol [175]. Compared to the nanoparticles such as liposomes, SLNs, and NLCs, preparation of nanoemulsion is simpler and less expensive; however, its addition to the food may alter organoleptic properties, potentially impacting consumer acceptance [167].

Lipid-based delivery systems are one of the main strategies for improvement of the solubility and bioavailability of water-insoluble antioxidants. They consist of biocompatible excipients that protect the encapsulated molecules from degradation during processing, storage, and digestion, while enabling improved dissolution in the GIT [176]. Nevertheless, despite their obvious advantages, lipid-based systems as delivery methods for lipophilic antioxidants are still not widely used in food technology. This is most likely due to the complexity and the high cost of manufacturing of such formulations [170]. Furthermore, the preparation of lipid-based delivery systems usually requires specialized equipment which then poses a challenge in the case of production scale-up. Also, it is important to take into consideration the influence of these forms on the appearance, smell, and taste of the food. Due to these limitations, some other formulation strategies for lipophilic antioxidants are being investigated, including the development of protein-based or polysaccharide-based delivery systems, the most common of which are CD-based formulations [167].

## 4. Cyclodextrin-Based Formulations of Lipophilic Antioxidants

As stated previously, CD encapsulation constitutes a pivotal strategy in the formulation of lipophilic compounds, notably antioxidants, by significantly enhancing their aqueous solubility, chemical stability, and bioavailability. When contrasted with alternative formulation approaches such as lipid-based nanoformulations—including liposomes and solid lipid nanoparticles—CD inclusion complexes present several fundamental advantages. Formulation of lipid-based carriers is often complex and expensive, requiring specialized equipment, which complicates the possibility of production scale-up. Additionally, their production necessitates the use of surfactants, organic solvents, and complex processing conditions that may raise concerns regarding biocompatibility and residual solvent toxicity. Conversely, CD complexation represents a simpler approach and typically proceeds via spontaneous, non-covalent interactions in aqueous media, circumventing the extensive use of potentially harmful solvents [177,178]. From an environmental and sustainability perspective, CD-based formulations offer substantial benefits since they are derived from renewable starch sources through enzymatic processes, ensuring their biodegradability and low ecological impact. CD complexation generally minimizes or eliminates the use of hazardous organic solvents, thereby reducing solvent waste and environmental emissions [179]. Furthermore, advancements in green chemistry have facilitated the development of environmentally benign synthesis methodologies for CD inclusion complexes. As explained previously, supercritical CO_2_ has emerged as a non-toxic solvent medium facilitating complex formation under mild conditions without solvent residues [51]. Other green solvents such as ionic liquids and deep eutectic solvents are under active investigation for their potential to further reduce the environmental footprint of complex preparation, and solid-state methods for the preparation of CD complexes are also emerging [52,53]. This aligns with principles of sustainable pharmaceutical and nutraceutical manufacturing, offering routes to minimize energy consumption and ecological burden without compromising product efficacy.

### 4.1. Cyclodextrin-Based Formulations of Carotenoids

Carotenoids are highly lipophilic compounds, soluble in organic solvents and insoluble in water. They are sensitive to pH, temperature, light, and oxygen [72]. When present within the food matrix in which they are synthesized, they are relatively stable, but once isolated, carotenoids become highly unstable, limiting their use as food ingredients due to degradation, colour changes, and loss of nutritional value [180]. To overcome such limitations and improve the stability, solubility, and bioavailability of carotenoids, CDs are used [72]. As mentioned previously, CDs are popular delivery systems, with a hydrophilic surface and a hydrophobic cavity, which makes them suitable for the encapsulation of lipophilic guest molecules. One of the newer delivery systems being used for carotenoids are nanosponges, which are mesh-like 3D structures with a hydrophobic interior and hydrophilic exterior, often formed by crosslinking CDs with various compounds [181]. Similarly to CDs, nanosponges improve the solubility, stability, and bioavailability of both water-soluble and lipid-soluble drugs.

Table 3 provides a detailed overview of recent studies on inclusion complexes formed between various carotenoids and CDs over the past decade. It summarizes the types of carotenoids examined, the types of CDs used, as well as the preparation methods applied. As shown in Table 3, βCD is one of the most commonly used CDs, and β-carotene is one of the most studied carotenoids. The primary objectives of the presented studies were to enhance solubility, improve stability (storage, heat, colour, and photostability), bioavailability, antioxidant activity, and other biological effects. Literature data show that CD complexation increased carotenoid solubility and slowed down the intestinal release of β-carotene in vitro, leading to improved bioaccessibility. Additionally, all conducted studies showed significantly improved stability of carotenoid-CD complexes, compared to native compounds (Table 3). On the other hand, CD complexation either increased or decreased measured antioxidant activity. For this purpose, various methodologies were employed by the authors, including simple in vitro assays (DPPH, TEAC, FRAP, CUPRAC), cytotoxicity testing, and animal studies (Table 3). Obtained results varied depending on guest, CD derivative, solvent/media, and applied assay. Variability of results in simple in vitro assays may arise from the antioxidant’s ability to interact with specific reagents in the reaction mixture, influenced by the polarity of the solvent or precise structure of the cyclodextrin complex. In cell-based or in vivo assays, factors such as the type of cell line used, gastrointestinal stability, or cell membrane permeability may affect results. Therefore, interpretation of these findings should be approached with caution. Given the well-documented limitations of in vitro and cell-based antioxidant assays—including their lack of physiological relevance and failure to account for absorption, metabolism, and bioavailability—it is essential to prioritize the findings of in vivo studies. Results from in vitro investigations should primarily be used to compare the relative antioxidant capacities of different cyclodextrin derivatives or methods of inclusion complex preparation and should not be extrapolated to predict in vivo effects.

As presented in Table 3, the inclusion complex of β-carotene and βCD prepared by co-precipitation, as well as the complex prepared by kneading and drying at 50 °C in 1:1, 1:2, and 1:3 molar ratios, showed more negative values of the zeta potential compared to free β-carotene, indicating that the formation of the inclusion complex led to an increased stability [182,191]. The solubility test of the β-carotene/βCD complex formed by extended co-precipitation showed twice the concentration (1.2 µg of β-carotene per mg of complex) compared to the complex formed by co-precipitation (0.6 µg of β-carotene per mg of complex) [186]. β-carotene encapsulated in three derivatives of βCD, methyl-β-cyclodextrin (MeβCD), HPβCD, and 2-hydroxyethyl-β-cyclodextrin (HEβCD), prepared by the precipitation method and vacuum drying, showed lower antioxidative and radical scavenging activity compared to free β-carotene. The highest antioxidant activity was observed in the complex with HEβCD and the lowest in the complex with MeβCD, which can be related to the deeper and more hydrophobic cavity. Çelik and co-workers and Puebla-Duarte and co-workers [72,191] reported similar antioxidant activity of the complex β-carotene/βCD compared to free β-carotene.

Regarding the inclusion complex of β-carotene and βCD, in vitro availability of β-carotene from the inclusion complex was investigated and evaluated through in vitro digestion, using rice as a food matrix. The complex was prepared by physical blending, kneading, and co-precipitation, as well as by co-precipitation followed by freeze-drying [179,181]. Unchanged or increased release of β-carotene was observed at particular stages of digestion, possibly due to the potential interference of rice as a food matrix. In vitro release from the inclusion complexes prepared with water-dispersible β-carotene extracted with absolute ethanol decreased consistently and was not influenced by the presence of rice as a food matrix, whereas hexane-soluble β-carotene extracted with ethanol:hexane mixture exhibited gradual release during intestinal digestion, but its release was reduced in the presence of rice as a food matrix [185,194]. They used three types of βCD-based nanosponges and reported improved in vitro release under physiological conditions. Furthermore, the complex exhibited an increased cytotoxic effect in both normal and kidney cancer cells compared to free β-carotene [194].

CD-based inclusion complexes with other types of carotenoids were investigated as well, including bixin, astaxanthin, fucoxanthin, lutein, zeaxanthin, and crocetin. Pinzon-Garcia and co-workers [190] investigated the effects of oral administration of the inclusion complex of bixin and βCD, prepared by mixing solutions and freeze-drying, on insulin resistance in high-fat-fed C57BL/6 obese mice. Oral administration of the inclusion complex not only resulted in a reduction in body weight, Lee’s index, and relative adipose tissue weight, but also significantly decreased serum cholesterol (CHT), triglycerides (TG), the CHT/HDL-c ratio, and glucose levels. The inclusion complexes of astaxanthin/MeβCD exhibited improved inhibition activity compared to pure astaxhantin, possibly due to enhanced solubility of astaxanthin and higher transport through the cell membrane [189]. Similar observations were made for fucoxanthin/HPβCD complex [192]. The investigation of applicability of lutein- and zeaxanthin-CD inclusion complexes showed improved solubility and permeability compared to native compounds. Among different tested CDs, RAMEB demonstrated the highest solubilizing efficiency, reaching solubility values of 572 ± 55 μM for lutein and 1470 ± 103 μM for zeaxanthin. Both in vitro and ex vivo porcine eye models showed that RAMEB-based formulations significantly enhanced the permeability of the carotenoid derivatives by increasing their solubility, while Raman mapping confirmed that CDs can enhance corneal permeability, even for highly lipophilic compounds [195].

Crocetin has been recognized as an active compound with pharmacological activities, particularly relevant for Alzheimer’s disease. γCD-based inclusion complex with crocetin was formed by dissolution and ultrasonic homogenization, followed by membrane extrusion. The use of γCD has facilitated the passage of crocetin through the blood–brain barrier, which makes it a potential delivery system to treat Alzheimer’s disease [196].

### 4.2. Cyclodextrin-Based Formulations of Tocopherols and Tocotrienols

Despite its recognized therapeutic potential, vitamin E (particularly α-tocopherol) is chemically labile, exhibiting high susceptibility to degradation under heat, light, oxygen exposure, and alkaline conditions [198]. Additionally, its strong lipophilicity leads to poor aqueous solubility, which limits bioavailability and poses significant challenges for formulation in both food and pharmaceutical applications [199]. Co-administration with dietary lipids has been shown to enhance intestinal absorption, likely through micelle-mediated transport mechanisms [200]. To overcome these physicochemical limitations—specifically poor water solubility and chemical instability—various esterified derivatives, such as tocopheryl acetate, have been developed. These derivatives offer improved oxidative and thermal stability and are less susceptible to isomerization [201]. However, recent evidence suggests that complexed free tocopherol, the natural and biologically active form, may exhibit greater stability than its esterified counterparts under certain conditions [202]. This may explain why most studies involving CD inclusion complexes have utilized tocopherol rather than tocopheryl acetate.

Several review articles have already summarized earlier studies, which predominantly involved simple CD-based formulations of tocopherols. For instance, López-Nicolás [203] reviewed the literature on complexes formed between CDs and various antioxidant compounds, including tocopherols. The review highlighted inconsistencies in the reported data, including conflicting findings on the antioxidant activity of host–guest complexes, variations in complexation constants for ostensibly identical systems, and differences in the bioavailability of antioxidant compounds upon complexation with CDs. The study also discussed recommendations regarding the use of natural versus chemically modified CDs. Subsequent reviews by Uekaji and co-workers and Wupper and co-workers [204,205] focused specifically on the influence of γCD on the bioavailability, stability, and bioactivity of nutraceutical compounds.

Different CDs were investigated for encapsulating tocopherols, including all native CDs, and their hydroxypropylated versions, as well as octenyl succinic βCD. In several studies, large ring CD were used which included up to 54 glucose units. More recent studies incorporated CDs in more complex formulations such as copolymers, Pickering emulsions, and hydrogels in order to improve tocopherol solubility/bioavailability (Table 4).

Native βCD and HPβCD are the most extensively studied derivatives for tocopherol inclusion. Iaconinotoand and co-workers [206] demonstrated that βCD–α tocopherol complexes prepared by kneading did not alter photostability or antioxidant activity, whereas HPβCD afforded improved storage stability at the expense of enhanced photostability. Conversely, Lange and co-workers [207] reported that lyophilized complexes of βCD and HPβCD markedly enhanced α-tocopherol thermal stability, with native βCD surpassing HPβCD, highlighting the critical influence of complexation methodology on protective efficacy. They also reported that the kneading method was ineffective for preparing the inclusion complex with the hydroxypropyl derivative of β-CD. This confirms that the method of complex preparation critically determines the protective effect of CD on tocopherol.

It seems that lyophilization enables more complete encapsulation, which results in enhanced thermal stability, whereas kneading may not sufficiently protect tocopherol during storage due to incomplete complexation. That is in line with studies comparing preparation methods for CD complexes that consistently report that lyophilization and solvent evaporation produce inclusion complexes with superior stability and dissolution properties compared to kneading [47]. Moreover, Celebioglu [208] showed improvement of stability, solubility, antioxidative activity, and photostability after electrospinning of inclusion complex between HPβCD without using a polymeric matrix to produce nanofibers. This simple approach could enable the usage of HPβCD for tocopherol applications in food, pharmaceuticals, and healthcare, thanks to the efficient antioxidant activity along with enhanced water solubility, prolonged shelf-life, and high photostability of Vitamin E.

Large-ring CDs (LRCDs) consisting of 22–54 glucose units and amphiphilic derivatives such as octenyl succinate/βCD have been explored for enhanced solubilization and emulsification of vitamin E. Kuttiyawong and co-workers [209] achieved an 800-fold increase in water solubility of vitamin E acetate using large ring-CD22–CD54 (10:1 ratio), albeit with a 30% reduction in radical-scavenging activity. Cao and co-workers [210], on the other hand, used LRCDs (C9–C22) and a completely different tocopherol:CD ratio (1:2) but also showed great potential of LRCD to improve the thermal stability and antioxidant activity of tocopherol. These studies could indeed serve as a reference for the more effective use of LRCDs as antioxidant carriers in the future. Recent studies employed octenyl succinic derivatives of βCD. In study of Ke and co-workers [211], encapsulation in octenyl succinic βCD enabled significantly better antioxidative activity of tocopherol, and it was able to improve the physical and oxidative stability of the emulsion, which is of great significance to the food industry since the complex addition to the emulsions significantly improved emulsifying properties (particle size distribution, z-potential, and creaming index).

As previously mentioned, βCD complexes with tocopherol were also used to form more complex formulations. Singh and co-workers [212] successfully formulated hyaluronic acid/βCD grafted copolymer to improve tocopherol solubility, while two recent studies also investigated different formulations with βCD as a component to examine formulation impact on tocopherol bioavailability in vivo. These highly valuable results confirmed that both approaches succeeded in improving tocopherol bioavailability.

Eid and co-workers [213] used an approach targeted towards the development of βCD hydrogel nanocomposites, which enhanced the controlled release and bioavailability of vitamin E. Additionally, the oral administration of these CDs with vitamin E to rats resulted in a sustained increase in the vitamin levels in plasma, even after 12 h post-administration, which proves the increase in the pharmacological bioavailability of vitamin E when incorporated into this type of delivery system [214]. However, no control sample using only βCD complex was included in the study.

Cheng and co-workers [215] formulated cinnamaldehyde/βCD Pickering emulsions and enhanced oral bioavailability of α-tocopherol in vivo. However, this study also did not include a control sample (pure α-tocopherol), and only the βCD complex was included.

**Table 4:** Cyclodextrin inclusion complexes with tocopherols (years 2015–2025).

Tocopherol	Cyclodextrin; Tocopherol:Cyclodextrin Ratio (molar)	Technology of Preparation	Characterization	Target Functionality Aspect	Effect	Reference
**alpha tocopherol**	βCD	kneading	NMR	Photostability	≡	[206]
				Stability	≡	
				Antioxidative activity	≡	
	HPβCD; 1:1			Photostability	↘ photostability	
				Stability	↗ stability	
				Antioxidative activity	≡	
	HPγCD			Photostability	↘ photostability	
				Stability	↗ stability	
				Antioxidative activity	≡	
	βCD; 1:1	freeze-drying	DSC, FTIR, NMR	Thermal stability	↗ thermal stability	[207]
	HPβCD; 1:1					
	HPβCD; 1:1; 1:2	nanofibres; electrospinning	NMR, XRD, FTIR, DSC	Stability	↗ stability	[208]
				Solubility	↗ solubility	
				Antioxidative activity	↗ antioxidative activity	
				Photostability	↗ photostability	
	osβCD; 1:12	freeze-drying	FTIR, NMR, SEM, AFM	Antioxidative activity	↗ antioxidative activity	[211]
				Emulsifying properties (particle size distribution, ζ-potential, and creaming index)	↗ emulsifying properties	
	LR-CD (CD9–CD22); 1:2	coprecipitation	SEM, FTIR, NMR	Thermal stability	↗ thermal stability	[210]
				Stability	↗ stability	
	Hyaluronic acid-βCD Grafted Copolymer	freeze-drying	FTIR, NMR, SEM, XRD,	Solubility	↗ solubility	[212]
	βCD Hydrogel nanocomposites; 1:8	coprecipitation; suspension chemical crosslinking	FTIR, DSC, XRD, SEM, TEM, AFM, CLSM	Bioavailavility	↗ bioavailability	[213]
	βCD/cinnamaldehyde based Pickering emulsions	freeze-drying; high speed shear emulsification	/	Bioavailavility	↗ bioavailability	[214]
**tocopheryl acetate**	βCD; 1:1	freeze-drying	IR, TGA, DSC	Stability	↗ stability	[202]
				Photostability	↗ photostability	
	LR-CD (CD22–CD54); 10:1	coprecipitation	FTIR	Solubility	↗ solubility	[209]
				Antioxidative activity	↘ antioxidative activity	

βCD (beta cyclodextrin); HPβCD (hydroxypropyl beta cyclodextrin); HPγCD (hydroxypropyl gamma cyclodextrin); osβCD (octenyl succinic beta cyclodextrin); LR-CD (large rig cyclodextrin); FTIR (Fourier transform infrared spectroscopy); TGA (thermogravimetric analysis); NMR (nuclear magnetic resonance); DSC (differential scanning calorimetry); XRD (X-ray Diffraction); SEM (scanning electron microscopy); AFM (Atomically Resolved Imaging); TEM (transmission electron microscopy); CLSM (Confocal Laser Scanning Microscopy); IR (Infrared Resonance); ≡ (no effect); ↗ (increased); ↘ (decreased).

### 4.3. Cyclodextrin-Based Formulations of Retinoids

Retinoids exhibit high sensitivity to light and heat, primarily due to their conjugated polyene structure. This configuration renders it susceptible to decomposition, structural rearrangement, and oxidative degradation in the presence of UV radiation and atmospheric oxygen [216]. Additionally, vitamin A demonstrates poor solubility and limited dispersibility in aqueous environments. These physicochemical limitations—namely, low water solubility and instability—significantly restrict its application in the food additive and cosmetics industries [199]. To address these issues, ester derivatives of vitamin A, such as retinyl acetate and retinyl palmitate, have been developed. These derivatives exhibit improved chemical and thermal stability and are less prone to oxidation and isomerization. Consequently, they are commonly employed as alternative sources of vitamin A. However, esterification does not alter the inherent polyenic structure of the molecule, and thus challenges related to stability and water solubility persist. To overcome these limitations, various strategies have been explored to enhance the stability of retinoids [216]. Among these, CD encapsulation has emerged as one of the most extensively studied approaches (Table 5).

Studies that investigated retinoid CD encapsulation usually focused on retinyl esters, acetate, and palmitate, and less frequently on retinol and retinoic acid. βCD, in particular, has garnered significant attention in presented studies, while simple CD derivatives such as HPβCD and HPγCD were used less frequently. More complex fatty amide-CD conjugates were also investigated. γCD was incorporated into metal–organic frameworks (MOFs), complex systems composed of metal clusters and organic linkers, as well as ternary complexes, were obtained using βCD and HPγCD and the amino acid arginine (Table 5).

Vilanova and co-workers [217] and Xu and Fathalla [218] successfully applied βCD for formation of inclusion complexes with retinoids. In all studies, a 1:1 molar ratio of retinoid to CD was used, except for retinol, which required a 1:2 ratio. Encapsulation led to enhanced solubility of the compounds, and, in Vilanova’s study [217], improved photostability was also observed.

Celebioglu and co-workers [219] demonstrated that electrospun inclusion complexes of retinyl acetate with HPβCD and HPγCD, prepared without a polymeric matrix, significantly improved the compound’s thermal stability, solubility, and antioxidant activity. Notably, the HPβCD complex exhibited superior solubilizing capacity, higher release efficiency, and stronger antioxidant potential compared to the HPγCD complex. Based on these findings, the authors proposed the use of electrospun CD-based nanofibers as a novel carrier system for food and dietary supplements, offering improved delivery and rapid oral dissolution characteristics.

More complex chemical systems involving covalently modified CDs—resulting in discrete or extended supramolecular entities—have also been employed for the encapsulation of retinoids. Kim and co-workers [220] developed amphiphilic βCD derivatives capable of forming micellar aggregates or vesicular structures, which are particularly advantageous for applications in surface activity enhancement, solubilization, and drug delivery. Their approach involved the covalent conjugation of fatty acids to the primary hydroxyl side of βCD, aiming to improve both the encapsulation efficiency and photostability of all-trans-retinol. Specifically, stearic and oleic acids were selected as model lipophilic moieties. The resulting mono-substituted CD derivatives—mono[6-deoxy-6-(octadecanamido)]-βCD and mono[6-deoxy-6-(octadecenamido)]-βCD—were shown to self-assemble into nano-vesicles in aqueous environments. These vesicles effectively encapsulated all-trans-retinol and significantly enhanced its stability against photo-degradation. Due to their amphiphilic nature and self-assembling capabilities, these modified CDs present a promising platform for retinoid delivery in both cosmetic and pharmaceutical formulations.

Zhang and co-workers [221] explored the use of MOF carriers for the stabilization and delivery of retinyl palmitate in food and pharmaceutical applications. For that purpose, MOFs were constructed using γCD as the organic ligand and potassium ions as the metal centres, forming highly porous, biocompatible materials suitable for bioactive compound delivery. Notably, retinyl palmitate encapsulated within the γCD-based MOFs (γCD/MOFs) exhibited significantly improved stability compared to commercial reference formulations, and an enhanced protective effect was attributed to the molecular conformation of retinyl palmitate within the MOF structure, which provided a physical barrier against environmental degradation.

In summary, CD encapsulation of different retinoids has been clearly shown to be an effective way to boost solubility and stability in vitro. The remaining task is to deepen our mechanistic understanding, demonstrate clear in vivo benefits, and develop scalable formulations. If these can be achieved, CD–retinoid complexes could advance to practical products for dermatology, ophthalmology, and nutrition.

### 4.4. Cyclodextrin-Based Formulations of Capsaicin, Coenzyme Q10, and Curcumin

CAP is the main active component of chilli peppers and is widely explored for pain management and as a dietary supplement. Its broader use as a dietary ingredient is limited by irritation of the GIT and high lipophilicity, which results in very low water solubility [222]. To address these limitations, advanced pharmaceutical formulations are being developed, including CD-based complexes (Table 6).

The effect of complexation on GI irritation was examined by Zhao and co-workers [223], who fed rats either free CAP or HPβCD/CAP. Histopathological analysis showed reduced gastric mucosal irritation with the complex, indicating its potential as a safer dietary formulation. The same study also demonstrated significantly higher bioavailability of HPβCD/CAP compared with free CAP, though human trials remain necessary. Chen and co-workers [224] evaluated CAP bioavailability following subcutaneous administration in rats. Plasma CAP was detectable at 10 min for HPβCD/CAP, compared with 80 min for free CAP, and overall bioavailability increased 2.36-fold. CAP is also used clinically for pain relief, either alone or as an adjuvant. Couto and co-workers [225] reported improved analgesia in mice when HPβCD/CAP was combined with mepivacaine; however, the study lacked a mepivacaine + free CAP group, preventing a direct comparison between the inclusion complex and pure CAP. To mitigate CAP cytotoxicity, Kadian and co-workers [226] developed βCD-based nanosponges for potential arthritis therapy. In vitro studies on macrophage cell lines showed reduced cytotoxicity relative to free CAP, alongside comparable anti-arthritic effects. Statistical comparisons with free CAP were not fully reported, leaving the magnitude of difference unclear.

Overall, these studies demonstrate that CDs can successfully form CAP inclusion complexes, with improved physicochemical properties, reduced GI irritation, and increased bioavailability.

The major pharmaceutical issue of CoQ10 delivery is its high molecular weight and poor water solubility, leading to poor oral bioavailability. To overcome these limitations, different advanced drug delivery systems were designed, with special emphasis on pharmacokinetic perspectives and clinical relevance. They include nanoparticles, solid dispersions, liposomes, nanoemulsions, self-emulsifying drug delivery systems, nanostructured lipid carriers, CDs, and nanocapsules [227]. CD-based formulations are presented in Table 6.

Gao and co-workers [228] explored αCD, βCD, γCD, and HPβCD complexes, followed by an in vivo dog study. Among the tested systems, γCD/CoQ10 showed the most favourable physicochemical profile. Beagle dogs administered γCD/CoQ10 exhibited significantly higher plasma CoQ10 levels than the γCD/CoQ10 physical mixture or free CoQ10, suggesting that γCD is promising for dietary formulations. Takahashi and co-workers [229] examined αCD, βCD, and γCD complexes in 20 fasting female volunteers. Bioavailability was assessed by plasma Cmax and AUC, again identifying γCD-CoQ10 as optimal. A separate clinical trial compared three CoQ10 formulations: βCD-CoQ10 liquid (commercial Q10VITAL^®^), βCD-CoQ10 powder, and CoQ10 in soybean oil. βCD-CoQ10 in liquid form produced the highest absorption and bioavailability, exceeding both the powder and oil formulations. CD complexation can also enhance CoQ10 stability. Fir and co-workers [230] reported that βCD and γCD complexes improved light and temperature resistance, with βCD showing superior photoprotection. Phase-solubility tests likewise indicated that βCD achieved stronger CoQ10 solubilization than γCD. Taken together, these findings support the use of CDs—especially γCD and βCD—as effective carriers to improve CoQ10 solubility, bioavailability, and stability.

CUR is another important lipophilic antioxidant, the primary bioactive in turmeric. It has anti-inflammatory and anticancer potential but suffers from high lipophilicity and low bioavailability. To overcome these limitations, it is often incorporated into advanced pharmaceutical formulations, including CD-based formulations (Table 6).

Paramera and co-workers [231] developed βCD/CUR complexes to evaluate stability under light, heat, and humidity, as well as behaviour in simulated gastric fluid (SGF) and simulated intestinal fluids (SIFs). The dissolution rates in SGF were high and the complex degraded in SIF, indicating it cannot withstand gastrointestinal conditions long enough to reach the small intestine intact for efficient absorption. Thermal stability also declined relative to free CUR, although preservation under elevated temperature and humidity was improved. Huand and co-workers [232] and Xu and co-workers [233] addressed these shortcomings using succinylated βCD complexes. Testing under natural light/temperature and SGF/SIF conditions revealed markedly improved storage, thermal, and photochemical stability, as well as retained gastrointestinal stability. This highlights how chemical modification of CDs can modulate complex properties. Furthermore, Song and co-workers [234] conducted an in vivo study in beagle dogs, showing that βCD/CUR achieved 231.9% relative oral bioavailability compared to free CUR. Zhang and co-workers [235] examined nasal delivery for Alzheimer’s therapy: in mice, CUR plasma and brain concentrations were 2.57-fold and 1.12-fold higher, respectively, compared to conventional CUR formulation, suggesting enhanced uptake.

Difluorinated curcumin (CDF) has also been tested. In mice, intravenous βCD/CDF improved bioavailability and pancreatic accumulation, while in vitro studies showed lower IC_50_ values in cancer cell lines. Zhang and co-workers [236] reported stronger antitumor activity of βCD/CUR compared to CUR in a mouse hepatoma model. Mechanistic studies in lung tumour cells indicated that βCD/CUR up-regulated p53/p21 pathway, down-regulated the CyclinE-CDK2 combination, and increased Bax/caspase 3 expression through the regulation of MAPK/NF-κB pathway, resulting in induced cellular apoptosis. Patro and co-workers [237] compared αCD, βCD, and γCD/CUR complexes in rabbits. Relative bioavailability was highest for αCD (460%) and βCD (365%), with γCD showing minimal improvement (99%). Advanced CD-based formulations continue to emerge. βCD nanosponges enhance solubility and stability, with crosslinker content strongly influencing properties [238], as do HPβCD and HPγCD/CUR nanofibers [239]. Celebioglu and co-workers demonstrated greater solubility, antioxidant activity, and complete disintegration in simulated saliva, supporting food or packaging applications.

γCD MOFs prepared by Sun and co-workers [240] exhibited good stability under light, heat, gastric pH (1.2), and PBS. Cai and co-workers [241] further evaluated γCD-MOF/CUR for fruit preservation, reporting improved light and oxygen barrier properties, though reduced water barrier capacity. Mechanical parameters (tensile strength, elongation at break) were favourable, and antibacterial activity with good biocompatibility was observed, leading to convincing evidence of enhanced fruit preservation. Nanotechnological approaches also improve anticancer efficacy. Wei and co-workers [242] demonstrated βCD nanoparticles reduced tumour volume and size in mice, reflecting a stronger tumour inhibition rate.

**Table 6:** Cyclodextrin inclusion complexes with capsaicin, CoQ, and curcumin (years 2015–2025).

Cyclodextrin	Technology of Preparation	Characterization	Target Functionality Aspect	Effect	Reference
**Capsaicin**					
**HPβCD**	Magnetic stirring	UV, IR, DSC	solubility, oral bioavailability, gastric irritation	↑ solubility ↑ oral bioavailability ↓ gastric irritation	[223]
**HPβCD**	freeze-drying	DSC, XRD, ITC, NMR SEM,	solubility	↑ solubility sustained release kinetics ↑ pain control in combination with mepivacaine compared to mepivacaine alone	[225]
**HPβCD**	saturation method	DSC, XRD	bioavailability, pharmacokinetics	↑ bioavailability faster absorption	[224]
**βCD nanosponges**	melt method, freeze-drying	UV, DSC, FTIR, XRD, NMR, RS, SEM, ELS	solubility, toxicity, anti-arthritic activity	↑ solubility ↓ toxicity = anti-arthritic activity	[226]
**Coenzyme Q10**					
**αCD** **βCD** **γCD** **HPβCD**	kneading method	XRD, DSC	oral bioavailability	↑ bioavailability of q10 in γCD complexes	[228]
**βCD** **γCD**	precipitation, freeze-drying	FTIR, DSC, TGA, XRD	solubility, thermal stability, photochemical stability	↑ solubility (γCD > βCD) ↑ thermal stability (βCD > γCD) ↑ photochemical stability (βCD > γCD)	[230]
**αCD** **βCD** **γCD**	high-pressure homogenization, spray drying	DSC	solubility, oral bioavailability	↑ solubility (γCD) ↑ bioavailability (γCD)	
**βCD (Q10VITAL^®^)**	precipitation, evaporation, thermal drying	DSC, XRD, IR	oral bioavailability	↑ bioavailability	[229]
**Curcumin**					
**βCD**	freeze drying, kneading, co-evaporation, co-precipitation	DSC	dissolution, thermal stability, photochemical stability, storage stability	↑ dissolution in SGF degradation of the complex in SPF ↓ photochemical stability ↑ thermal stability ↑ storage (humidity) stability	[233]
**sβCD**	freeze-drying	XRD, TGA, FTIR, NMR	solubility, photochemical, thermal, storage, gastrointestinal stability	↑ solubility ↑ storage stability ↑ thermal stability ↑ photochemical stability ↑ stability in gastrointestinal conditions	[232]
**sβCD/chitosan**	freeze-drying	XRD, TGA, FTIR, NMR	gastrointestinal stability, controlled release	↑ solubility ↑ stability in gastrointestinal conditions	[233]
**αCD** **βCD** **γCD**	pulverization	FTIR, DSC, XRD, NMR	solubility, oral bioavailability	↑ solubility ↑ bioavailability in order αCD > βCD > γCD	[237]
**βCD**	freeze-drying	FTIR	anticancer activity (liver, lungs)	↑ anticancer activity	[236]
**βCD**	co-precipitation, freeze-drying	FTIR, XRD	nasal bioavailability, in vitro cellular uptake	↑ bioavailability ↑ cellular uptake	[235]
**βCD**	precipitation	FTIR, XRD, SEM	oral bioavailability	↑ bioavailability	[234]
**βCD**	Kneading	FTIR, DSC, XRD, NMR, SEM, CVS	solution, intravenous bioavailability, anticancer activity in pancreas	↑ solubility ↑ bioavailability and concentration in pancreas ↑ anticancer activity in vitro	[243]
**βCD** **βCD nanosponges**	sonication, solvent-free melting method (nanosponge)	DSC, XRD, FTIR, SEM	solubility, dissolution	↑ solubility =dissolution rate	[238]
**γ-MOF**	impregnation	XRD, FTIR	Thermal stability, photochemical stability, pH stability	↑ thermal stability ↑ photochemical stability ↑ stability under different pH	[240]
**γ-MOF composite films**	vapour diffusion (multiple step preparation)	NMR, FTIR, XRD	mechanical properties, barrier properties, antioxidative activity, antimicrobial activity, biocompatibility, fruit preservation	↑ light barrier ↑ mechanical properties ↓ water barrier ↑oxygen barrier significant antibacterial activity good biocompatibility (80% zebrafish survival) ↑ fruit preservation	[241]
**HPβCD** **HPγ nanofibers**	electrospinning	NMR, FTIR, XRD, DSC, TGA	solubility, dissolution, antioxidative activity	↑ solubility (HPγCD) complete disintegration of fibre structure and dissolution in SSF ↑ antioxidative activity (HPγCD)	[239]
**βCD nanoparticles**	freeze-drying	NMR, FTIR, DSC, XRD, DLS; TEM	anticancer activity	↑ anticancer activity	[242]

αCD (alpha-cyclodextrin); βCD (beta-cyclodextrin); γCD (gamma cyclodextrin); HPβCD (hydroxypropyl-beta-cyclodextrin); sβCD (succinylated-beta-cyclodextrin); FTIR (Fourier transform infrared spectroscopy); SEM (scanning electron microscopy); DSC (differential scanning calorimetry); NMR (nuclear magnetic resonance spectroscopy); ELS (electrophoretic light scattering); IR (Infrared Resonance); XRD (X-ray diffraction study); TGA (thermogravimetric analysis); ITC (isothermal titration calorimetry); CVS (cyclic voltametric study); RS (Raman spectroscopy); UV-VIS (Ultraviolet-Visible Spectroscopy); MOF (metal–organic framework); SGF (simulated gastric fluid); SPF (simulated pancreatic fluid); ↑ (increased); ↓ (decreased); = (similar effect).

## 5. Conclusions

CD-mediated formulation of lipophilic antioxidants represents a promising strategy to overcome intrinsic limitations of lipophilicity and chemical instability and offers significant advantages over alternative formulation strategies, considering different aspects of sustainability. Selection of CD type, chemical modification, host–guest stoichiometry, and complexation technique profoundly influences the physicochemical properties and functional performance of the resulting inclusion complexes. Future work should prioritize systematic head-to-head comparisons—including cost–benefit analyses—between native, modified, and large-ring CDs, as well as between simple inclusion complexes versus advanced polymeric or particulate formulations, to guide the rational design of effective and scalable nutraceutical products.

## Figures and Tables

**Table 1:** Physicochemical properties, safety, and application of pharmaceutically relevant cyclodextrins [13,14,15,16,17].

CD	Number of Glucopyranose Units	MW (g mol^−1^)	Aqueous Solubility (mg/mL)	Inner Cavity Diameter (Å)	Toxicity (LD_50_ or NOEL)	Application
α-cyclodextrin (αCD) [10,12]	6	972	145	4.5–5.3	Low LD50 1000 mg/kg; rat; IV route LD50 > 5000 mg/kg; rat; oral route	Food additive, oral and parenteral pharmaceuticals
β-cyclodextrin (βCD) [9,10,12,13]	7	1135	18.5	6.0–6.5	Moderate (nephrotoxicity) LD50 788 mg/kg; rat; IV route LD50 > 5000 mg/kg; rat; oral route	Food additive, drug solubilization and stabilization in oral and dermal pharmaceuticals, cosmetics
Hydroxypropyl-β- cyclodextrin (HPβCD) [9]	7	1400	>600	5.8–6.5	Very low NOEL 3600 mg/kg/day; rat; oral route	Drug solubilization and stabilization in oral, ophthalmic, and parenteral pharmaceuticals, cosmetics
Randomly methylated β-cyclodextrin (RAMEB) [9,13]	7	1312	>500	5.8–6.5	High (haematolysis, cytotoxicity) LD50 > 8000 mg/kg; rat; oral route NOEL 300 mg/kg/day; rat; oral route	Research, topical drug delivery (nasal and ophthalmic)
Sulphobuthylether-β-cyclodextrin (SBEβCD) [9,13]	7	2163	>500	7.5–8.3	Low NOEL 500 mg/kg/day; rat; oral route	Solubilization and stabilization of parenteral pharmaceuticals
γ-cyclodextrin (γCD) [10,11]	8	1297	232	7.5–8.3	Low LD50 > 3750 mg/kg; rat; IV route LD50 > 8000 mg/kg; rat; oral route	Solubilization in oral and parenteral pharmaceuticals
Hydroxypropyl-γ-cyclodextrin (HPγCD) [10,11]	8	1576	>500	7.5–8.3	Low LD 50 and NOEL not available	Solubilization of ophthalmic and parenteral pharmaceuticals

LD_50_—the amount of a material, given all at once, which causes the death of 50% (one half) of a group of test animals; NOEL (no observable effect level)—the highest dose or exposure level of a substance or material that produces no noticeable (observable) toxic effect on tested animals.

**Table 2:** Health-promoting effects of oral supplementation of antioxidants.

Compound	Health Effect	Outcomes	Participants	Study Design	Dosage/Duration	Reference
**Carotenoids**						
**Lutein**	brain protection	cognitive function improvement improvement in visual episodic memory in young and middle-aged people favourable effect on grey matter volumes, resting-state connectivity, and learning activity in healthy older individuals	healthy young (≥18 years), middle-aged (40–60 years), or older (≥60 years)	randomized controlled trials (systematic review)	10 mg lutein daily/12 months	[98,99]
	eye protection	MPOD ^a^, visual sensitivities of early AMD ^b^ patients increasement	early AMD ^b^ patients adults (≥40 years)	randomized, double-blinded, placebo-controlled trial, meta-analysis	10 mg daily/2 years 20 mg daily/6 months	[100,101]
	anti-inflammatory	better antioxidant capability to reduce inflammatory response and lipid peroxidation	adults (<50 years)	meta-analysis	>10 mg daily/<12 weeks	[102]
	skin protection	better defence against UVA/B and UVA1-induced cutaneous gene expression	adults	placebo-controlled, double-blinded, randomized	10 mg daily/12 weeks	[103]
**Lutein (L) and zeaxanthin (Z)**	eye protection	MPOD ^a^ increasement higher gains in the tear film break-up time, photo-stress recovery time	healthy adults (>18 years) adults (18 to 65 years) who spent more than six hours a day in front of an electronic screen	meta-analysis randomized, double-blind, placebo-controlled study	>10 mg/day L + Z, ≥3 weeks 10 mg L + 2 mg Z/6 months	[104,105]
	neuroprotective/neurocognitive	improvement in composite memory (only male), cognitive flexibility, complex attention MPOD ^a^ increasement complex attention, spatial memory, and reasoning ability improvement cerebral circulation supports neurocognitive function improvement	healthy adults (average 72–74 years) healthy young adults (18–30 years)	randomized, double-blind, placebo-controlled trial	10 mg L + 2 mg Z daily/12 months	[106,107,108]
**Lycopene**	cardioprotective	better adjustment of cardiovascular parameters and inflammatory conditions in CVD ^c^ patients possible health advantages in the treatment of heart disease	CVD ^c^ patients (45–73 years) healthy individuals (≥60 years)	randomized controlled study, meta-analysis	7 mg daily/1 month >15 mg/daily (up to 30 mg/daily)/<12 weeks	[109,110]
	antihypersensitive	efficacy in lowering SBP ^d^ in hypertensive individuals	hypertensive individuals (35–60 years)	double blind, randomized dose–response study	15 and 30 mg/4 weeks	[111]
	skin protection	better defence against UVA/B and UVA1-induced cutaneous gene expression	adults	placebo-controlled, double-blinded, randomized trial	5 mg daily/12 weeks	[103]
**Astaxanthin**	neuroprotective	improvement in verbal fluency, recall after 5 min + cued recall, instantaneous recall, and Stroop test score	healthy adults (45–64 years)	randomized, double-blind, placebo-controlled trial	8 mg daily/2 months	[112]
	anti-obesity	inflammatory cytokines decrease, better lipid profiles, and lessened hemostatic problems	diabetic patients, type 2 (40–75 years)	double-blind, placebo-controlled study	6 and 12 mg daily/8 weeks	[113]
	anti-inflammatory	lower levels of muscle enzymes in plasma blunted systemic inflammatory response	male football players	randomized, double-blind and placebo-controlled study	4 mg daily/12 weeks	[114]
		environmental damage-induced skin deterioration prevention	healthy women (35–60 years)	randomized double-blind, parallel-group, placebo-controlled study	6 and 12 mg daily/16 weeks	[115]
		CRP ^e^ decreasement	healthy individuals (<50 years)	meta-analysis	2 to ≤10 mg daily/<12 weeks	[102]
**β-Cryptoxanthin**	antioxidative	biological parameters improvement in patients with NAFLD ^f^	NAFLD ^f^ patients	clinical trial	3 mg/12 weeks	[116]
**Vitamin E (Tocopherols and Tocotrienols)**						
**Vitamin E**	antioxidative	effective in reducing itching and lesions in AD ^g^ patients, lessen oxidative stress conditions, and relieve clinical symptoms in people with knee osteoarthritis	patients with AD ^g^ (10–50 years) ≥18-year-old adults, clinically diagnosed with knee osteoarthritis	double-blind, randomized, placebo-controlled trial, randomized controlled study	400 IU daily/4 months 400 IU daily/2 months	[117,118]
	anti-inflammatory	lessen oxidative stress in people with type 2 diabetes during fasting and after meals	patients with type 2 diabetes	single-blind placebo-controlled trial	400 IU daily/6 weeks	[119]
**Retinoids**						
**Isotretinoin**	anti-inflammatory	stabilization of symptoms, reduction in hyperpigmentation level, and slowing the progression of the disease	patients with LPP ^h^	prospective study	20 mg daily/6 months	[120]
		efficient therapy for mild to severe acne vulgaris	patients with moderate to severe acne vulgaris	prospective randomized comparative study	20 mg/24 weeks	[121]
		sufficient healing for the symptoms of PCOS ^i^ with severe cystic acne (who are not suitable candidates for OCP ^j^ use)	patients with PCOS ^i^ and acne (18–40 years, BMI 18–44 kg/m2)	prospective study	20–40 mg daily	[122]
**Others**						
**Capsaicin**	ergogenic	improvement in middle distance running (1500 m) performance and reduced ratings of perceived exertion in physically active adults, extended the time until fatigue without altering energy expenditure, oxygen consumption, lactate, and the rate of perceived effort, acutely enhanced muscular endurance, and reduction in the rate of perceived exertion	physically active adults (20–35 years) healthy adults	randomized, double-blind, crossover design, meta-analysis	12 mg 45 min before exercises	[123,124,125]
**Curcumin**	cardioprotective	improvement in endothelial function	healthy adults (19–29 years)	controlled double-blind parallel prospective study	200 mg curcumin in the form of CurcuWIN (curcumin + demethoxycurcumin + demethoxycurcumin)/8 weeks	[126]
		resistance and conduit artery endothelial function improvement	healthy men and postmenopausal women (45–74 years)	double-blind, parallel design, randomized study	2000 mg daily Longvida^®^ (~400 mg curcumin)/12 weeks	[127]
	antioxidative	antioxidant capacity improvement in individuals with diabetes, proteinuric CKD ^k^ reduction in lipid peroxidation in plasma in patients with nondiabetic proteinuric CKD ^k^	nondiabetic or diabetic proteinuric CKD ^k^	randomized double-blind placebo-controlled clinical trial	320 mg daily/8 weeks	[128]
		MDA ^l^ concentration reduction and increase in total antioxidant capacity	adults (average 27.60 ± 3.79 years)	meta-analysis	645 mg daily/67 days	[129]
	anti-inflammatory	considerable reduction in patients’ dyslipidaemia lowering serum levels of triglycerides, LDL ^m,^ and VLDL ^n^ cholesterol blood lipid levels regulation	patients with coronary artery disease	randomized double-blind placebo-controlled trial	500 mg capsules, four times a day/8 weeks	[130]
**Coenzyme Q10**	cardioprotective	statin-associated muscle symptoms decrease, additional strategy to treat statin-induced myopathy	patients with cardiovascular diseases	meta-analysis	100 to 600 mg daily/30–90 days	[131]
		improvement in ejection fraction larger advantages for people with heart failure as opposed to those with other cardiovascular conditions	adults (18 and older) with or without heart failure or cardiovascular diseases	meta-analysis	>200 mg/day, more than 12 weeks	[132]
	antioxidative	oxidation decreasement	healthy, trained firemen (38.9 ± 8.7 years)	randomized, double blind, and placebo-controlled trial	200 mg daily/2 weeks before intense physical activity	[133]
	anti-inflammatory	fatigue and depression improvement in patients with MS ^o^	patients with MS	randomized, double-blinded, placebo-controlled trial	500 mg daily/12 weeks	[134]
		lowering fibromyalgia patients’ pain, exhaustion, and sleep disturbance decrease in severity, duration, and migraine frequency	fibromyalgia patients non-menopausal women with episodic migraine diagnosed between the ages of 18 and 50	randomized, open-label, crossover study randomized, double-blind, placebo-controlled clinical trial	200 mg/2 times a day/3 months	[135,136]

^a^ MPOD—macular pigment optical density; ^b^ AMD—age-related macular degeneration; ^c^ CVD—cardiovascular diseases; ^d^ SBP—systolic blood pressure; ^e^ CRP—C-reactive protein; ^f^ NAFLD—nonalcoholic fatty liver disease; ^g^ AD—atopic dermatitis; ^h^ LPP—Lichen planus pigmentosus; ^i^ PCOS—polycystic ovary syndrome; ^j^ OCP—oral contraceptive pills; ^k^ CKD—chronic kidney disease; ^l^ MDA—malondialdehyde; ^m^ LDL—low density lipoproteins; ^n^ VLDL—very low density lipoproteins; ^o^ MS—multiple sclerosis.

**Table 3:** Cyclodextrin inclusion complexes with carotenoids.

Carotenoid	Cyclodextrin	Technology of Preparation	Characterization	Target Functionality Aspect	Effect	Reference
**β-carotene**	**MeβCD** **HPβCD HeβCD**	precipitation and vacuum drying	FTIR	phase solubility, antioxidant activity	↑ solubility ↓ antioxidant activity ↓ radical scavenging activity	[182]
**β-carotene**	**βCD**	physical blending, kneading, and co-precipitation	SEM, FTIR	antioxidant activity, in vitro release	↓ release of β-carotene ↑ antioxidant activity	[183]
***all-[E]*- and *[Z]*-lycopene, β-carotene**	**αCD** **βCD** **γCD**	dissolution, stirring, nitrogen sparging for emulsion formation, and freeze-drying	SEM, LCSM, FTIR, DSC	stability, antioxidant activity	↓ degradation of carotenoids ↑ antioxidant activity (αCD and βCD)	[184]
**β-carotene**	**βCD**	co-precipitation and freeze drying	SEM, DSC, FTIR	in vitro release properties (extractability of β-carotene)	↓ in vitro release	[185]
**β-carotene**	**βCD**	co-precipitation and extended co-precipitation	FTIR, NMR, TEM	solubility	↑ solubility	[186]
**red bell pepper carotenoids**	**HPβCD**	ultrasound homogenization, stirring, centrifugation, and freeze drying (complex of carotenoids and 2-HPβCD in 1:4, 1:6, 1:8, and 1:10 mass ratio)	FTIR, DSC, DLS, ^1^H NMR	solubility	↑ solubility	[187]
**yellow bell pepper carotenoids**	**βCD**	ultrasonic homogenization and kneading	FTIR, DSC, HPLC	colour stability of isotonic drinks	↑ colour stability	[188]
**astaxanthin**	**MeβCD**	physical blending, kneading, and spray drying (1:2 molar ratio)	FTIR, DSC, ^1^H NMR, UV, XRD	in vitro evaluation (solubility, bioaccessibility, antioxidant activity, antiproliferative activity)	↑ solubility ↑ dissolution study ↑ bioaccessibility ↑ radical scavenging activity ↑ inhibition activity	[189]
**bixin**	**βCD**	mixing solutions and freeze drying	FTIR, XRD, TG/DTG-DTA, ^1^H NMR, 2D ROESY	insulin resistance, insulin-stimulated glucose uptake, effect of a high-fat diet with and without treatment compared with the normal diet, effect of treatment on adiposity and on liver in high-fat fed C57BL/6 obese mice (body weight, Lee’s Index, adiposity, CHT, TG, CHT/HDL-c, glucose levels (metabolic markers) and liver markers (AST and ALT) were determined)	↓ metabolic and liver parameters ↓ body weight ↓ Lee’s index ↓ relative adipose tissue weight ↓ serum cholesterol (CHT) ↓ triglycerides (TG) ↓ CHT/HDL-c ratio ↓ glucose levels	[190]
**β-carotene**	**βCD**	co-precipitation (40:60 (% *w*/*w*))	UV, FTIR, Raman spectroscopy, DSC, TGA, SEM, XRD	stability, antioxidant activity, bioavailability	↑ stability = antioxidant activity erythroprotective effect	[191]
**fucoxanthin**	**HPβCD**	mixing solutions, blending, ultrasound homogenization, stirring, evaporation, and lyophilization	UV, FTIR, SEM, XRD, DSC	solubility, heat, storage, and gastrointestinal stability, antiproliferative activity	↑ solubility ↑ stability ↑antitumor activity	[192]
**lycopene**	**βCD**	co-precipitation and drying at 50 °C	SEM, UV, HPLC, DSC	solubility, thermal and photostability, antioxidant activity	↑ solubility ↑ thermal and photostability ↑ antioxidant activity	[193]
**β-carotene**	**βCD**	nanosponges with 2 cross-linkers (EPI-2:1 and HMDI-4:1)	UV-VIS, FTIR, DSC, XRD, FE-SEM	solubility, in vitro release, storage, photostability, cytotoxicity	↑ solubility (EPI as cross-linker) ↑stability ↑ in vitro release (HMDI as cross-linker) ↑ cytotoxicity (HMDI as cross-linker)	[194]
**lutein, zeaxanthin**	**HPβCD** **HPγCD, RAMEB**	dissolution and stirring	^1^H NMR	solubility, stability, in vitro corneal permeability, ex vivo corneal permeability, and mapping of the excised porcine corneas	↑ permeability ↑ corneal permeability	[195]
**β-carotene**	**βCD**	kneading (1:1, 1:2 and 1:3 molar ratios) and drying at 50 °C for 24 h	FTIR, DSC	stability, solubility, in vitro dissolution study	↑ stability ↑ solubility (1:2) ↑ in vitro dissolution	[182]
**crocetin**	**γCD**	dissolution, ultrasonic homogenization, neutralization, pH adjustment, and membrane extrusion	FTIR, DSC	therapeutic efficacy, cell viability, cytotoxicity, pharmacokinetic study, antioxidant activity, bioavailability	↑ solubility ↓ expression of CTFs and levels of Aβ ↑ neuroprotective effect ↑antioxidant activity ↑ absorption (intravenous injection) ↑ bioavailability ↑ passage of the crocetin through the blood–brain barrier	[196]
**lutein**	**βCD**	spray-drying	SEM, FTIR, DSC	saturation solubility, bioaccessibility	↑ solubility ↑ bioaccessibility	[197]

αCD (alpha-cyclodextrin); βCD (beta-cyclodextrin); γCD (gamma cyclodextrin); MEβCD (methyl-beta-cyclodextrin); HPβCD (hydroxypropyl-beta-cyclodextrin); HEβCD (hydroxyethyl-beta-cyclodextrin); RAMEB (randomly methylated beta-cyclodextrin); FTIR (Fourier transform infrared spectroscopy); SEM (scanning electron microscopy); LCSM (laser confocal scanning microscopy); DSC (differential scanning calorimetry); NMR (nuclear magnetic resonance spectroscopy); TEM (transmission electron microscopy); DLS (dynamic light scattering); ^1^H NMR (proton nuclear magnetic resonance spectroscopy); XRD (X-ray diffraction study); TGA (thermogravimetric analysis); 2D ROESY (two-dimensional NMR spectra); FE-SEM (field emission scanning electron microscopy); ↑ (increased); ↓ (decreased); = (similar effect).

**Table 5:** Cyclodextrin inclusion complexes with carotenoids (years 2015–2025).

Retinoid	Cyclodextrin; Retinoid:Cyclodextrin Ratio (molar)	Technology of Preparation	Characterization	Target Functionality Aspect	Effect	Reference
**Retinyl palmitate**	βCD; 1:1	freeze-drying	FTIR, TGA	Solubility	↗ solubility	[217]
				Photostability	↗ photostability	
**Retinol**	βCD; 2:1	coprecipitation; freeze-drying	FTIR, NMR	Solubility	↗ solubility	[197]
**Retinyl acetate**	βCD; 1:1					
**Retinyl palmitate**	βCD; 1:1					
**Retinoic acid**	βCD; 1:1	kneading	DSC, FTIR	Solubility	↗ solubility	[218]
	HPβCD; 1:1					
**Retinyl acetate**	HPβCD; 1:2	nanofibres; electrospinning	NMR, XRD, FTIR, DSC, TGA	Thermal stability	↗ thermal stability	[219]
				Solubility	↗ solubility	
				Antioxidative activity	↗ antioxidative activity	
	HPγCD; 1:2			Stability	↗ stability	
				Solubility	↗ solubility	
				Antioxidative activity	↗ antioxidative activity	
**Retinol**	sβCD	coprecipitation	FTIR, TGA	Photostability	↗ photostability	[220]
	oβCD					
**Retinyl palmitate**	γCD-MOF; 1:3.2	coprecipitation	XRD	Stability	↗ stability	[221]

βCD (beta cyclodextrin); HPβCD (hydroxypropyl beta cyclodextrin); HPγCD (hydroxypropyl gamma cyclodextrin); sβCD (Mono[6-Deoxy-6-(Octadecanamido)] beta cyclodextrin, stearamido beta cyclodextrin); oβCD (Mono[6-Deoxy-6-(Octadecenamido)] beta cyclodextrin, Oleamido beta cyclodextrin; γCD-MOF (gamma cyclodextrin metal–organic frameworks); FTIR (Fourier transform infrared spectroscopy); TGA (thermogravimetric analysis); NMR (nuclear magnetic resonance); DSC (differential scanning calorimetry); XRD (X-ray Diffraction); ↗ (increased).

## Data Availability

No new data were created or analyzed in this study. Data sharing is not applicable to this article.

## Abbreviations

The following abbreviations are used in this manuscript:

CD	cyclodextrin
αCD	α-cyclodextrin
βCD	β-cyclodextrin
γCD	γ-cyclodextrin
HPβCD	hydroxypropyl-β-cyclodextrin
RAMEB	randomly methylated β-cyclodextrin
SBEβCD	sulphobuthylether-β-cyclodextrin
HPγCD	hydroxypropyl-γ-cyclodextrin
MeβCD	methyl-β-cyclodextrin
HEβCD	hydroxyethyl-β-cyclodextrin
LD	lethal dose
NOEL	no observed effect level
FDA	Food and Drug Administration
USP/NF	United States Pharmacopeia and the National Formulary
DSC	differential scanning spectroscopy
XRPD	X-ray powder diffraction
ssNMR	solid state NMR
SOD	superoxide dismutase
ROS	reactive oxygen species
CoQ10	coenzyme Q10
GIT	gastrointestinal tract
SLN	solid lipid nanoparticle
NLC	nanostructured lipid carrier
LRCDs	large-ring cyclodextrins
CAP	capsaicin
CUR	curcumin
SGF	simulated gastric fluid
SIF	simulated intestinal fluid
MOF	metal–organic framework
